# What do young adolescents think about taking part in longitudinal self-harm research? Findings from a school-based study

**DOI:** 10.1186/s13034-018-0230-7

**Published:** 2018-05-02

**Authors:** Joanna Lockwood, Ellen Townsend, Leonie Royes, David Daley, Kapil Sayal

**Affiliations:** 1 Division of Psychiatry & Applied Psychology, Institute of Mental Health, University of Nottingham, University of Nottingham Innovation Park, Triumph Road, Nottingham, NG7 2TU UK; 2 Centre for ADHD and Neurodevelopmental Disorders Across the Lifespan, Institute of Mental Health, University of Nottingham, Nottingham, UK; 3 Self-Harm Research Group, School of Psychology, University of Nottingham, Nottingham, UK

**Keywords:** Self-harm, Adolescence, Ethics, Longitudinal, Multi-methods, Mood-mitigation

## Abstract

**Background:**

Research about self-harm in adolescence is important given the high incidence in youth, and strong links to suicide and other poor outcomes. Clarifying the impact of involvement in school-based self-harm studies on young adolescents is an ethical priority given heightened risk at this developmental stage.

**Methods:**

Here, 594 school-based students aged mainly 13–14 years completed a survey on self-harm at baseline and again 12-weeks later. Change in mood following completion of each survey, ratings and thoughts about participation, and responses to a mood-mitigation activity were analysed using a multi-method approach.

**Results:**

Baseline participation had no overall impact on mood. However, boys and girls reacted differently to the survey depending on self-harm status. Having a history of self-harm had a negative impact on mood for girls, but a positive impact on mood for boys. In addition, participants rated the survey in mainly positive/neutral terms, and cited benefits including personal insight and altruism. At follow-up, there was a negative impact on mood following participation, but no significant effect of gender or self-harm status. Ratings at follow-up were mainly positive/neutral. Those who had self-harmed reported more positive and fewer negative ratings than at baseline: the opposite pattern of response was found for those who had not self-harmed. Mood-mitigation activities were endorsed.

**Conclusions:**

Self-harm research with youth is feasible in school-settings. Most young people are happy to take part and cite important benefits. However, the impact of participation in research appears to vary according to gender, self-harm risk and method/time of assessment. The impact of repeated assessment requires clarification. Simple mood-elevation techniques may usefully help to mitigate distress.

## Background

Self-harm, here defined as any act of self-poisoning or self-injury irrespective of motivation or suicidal intent [1], is a common and significant health concern in adolescence. Average lifetime prevalence of self-harm in community-based samples of adolescents in Europe and Australia has been estimated at 17.8% [2], with rates comparable internationally [3]. While self-harm for many is about preserving rather than ending life [4] it is nonetheless strongly linked to completed suicide, with 40–60% of those who die by suicide having a history of self-harm [5]. Youth who self-harm are also at increased risk of mental health difficulties and multiple life problems such as increased alcohol use and relationship difficulties [6, 7]. Adolescents who self-harm thus represent an extremely vulnerable group.

Adolescence—the developmental period spanning 12–25 years of age—is an important time to focus research on self-harm as these years are likely to include the onset (12–14 years), peak (15–24 years) and start of remittance of the behaviour [8–10]. Rates of self-harm behaviour are three times higher in adolescents than adult populations [11]. Much self-harm research to date has focused on mid to late adolescence. This approach is important given high rates of self-harm in this age group [12], but this focus may also be a consequence of the additional ethical and procedural challenges involved in research with younger age groups, and a reluctance on the part of ethics committees and Institutional Review Boards (IRBs) to sanction self-harm research in those perceived to be at heightened vulnerability. Yet, research at earlier stages of adolescence is important to understand how and why self-harm first develops [13]. Moreover, recent reports suggest that increasing rates of self-harm across adolescence show the steepest rise in girls under 16 years of age [14], suggesting that early adolescence is a period of particular concern in adolescent self-harm. Most young people who self-harm do not seek clinical support [2], and this is particularly the case in young adolescents (aged 12–14 years) where community-based cases of self-harm outnumber hospital presentations by up to 20 times [15]. School-based studies thus provide a vital opportunity to engage with an early adolescent population at risk of self-harm who may otherwise remain hidden. Work which strengthens the evidence base for the ethical suitability of self-harm studies in younger age groups in school-based samples can help to reframe the calculation of risk for future research in this critical area.

### Ethical challenges—overstated risks?

For researchers and regulatory bodies rightfully mindful of the need to balance the delivery of research objectives against ensuring participant wellbeing [16, 17], a key concern is that asking participants about self-harm/suicidality may introduce, reinforce or exacerbate such acts, or cause undue psychological distress [16]. In fact, reviews of the evidence, which have pooled findings across adult and adolescent populations, have suggested that asking about such issues is not associated with negative outcomes [18, 19] and may, in fact, confer benefits for those at most risk [20]. This is important for anonymous survey-based studies where a direct gauging of impact is impossible.

### Response from school-based youth to self-harm studies

Relatively few studies have sought to understand the impact that being asked specifically about self-harm has on school-based respondents. Hasking and colleagues [21] examined whether completing a survey about non-suicidal self-injury (NSSI), suicidality, and wider psychological constructs was perceived as either enjoyable or upsetting/worrying, in school-based students aged 12–18 years. Overall, the majority of participants enjoyed participation at baseline and at 1-year follow-up with only a minority finding participation to be upsetting/worrying, but those who had thought about or experienced self-harm were more likely to have had this response. Notably, Hasking and colleagues found that girls were more likely than boys to find the survey upsetting, but also more likely than boys to report enjoying participation. There may be a nuanced gendered distinction in reactions to sensitive research that warrants further analysis. It is important, given the greater prevalence of self-harm in girls relative to boys [14], to establish further if this gendered distinction is moderated by the likelihood that an individual has a history of self-harm i.e. whether vulnerability is conferred by self-harm status, by gender, or an interaction between the two. Other school-based studies have similarly found that while overall participation in a research survey is viewed positively there are nonetheless links between increased vulnerability and likelihood of reporting distress [22, 23]. Importantly, these studies point to factors such as being “interested” in the topic [22] or finding it “worthwhile” [23] which partially mitigate this distress, and similar findings have been found in a study with young adults [24]. Notably, one of these studies only included boys from a select-entry school [22] which limits how generalisable these findings are to a general school population; the other [21], gathered reactions to questions on suicide, drug use and sexual abuse, issues which could arguably have a different personal resonance than self-harm in a younger population. Nonetheless these studies suggest that there may be an important distinction when making a judgment of impact in self-harm research, between having an emotional response and a cognitive evaluation of that response, and highlight that more evidence, particularly examining gender differences is now needed.

### Establishing short-term risk

Not all studies have found that those at highest risk are more likely to experience distress. In suicide research [20], high risk students with raised depressive symptomatology who answered survey questions about suicide were less likely to report distress or suicidality immediately afterwards and 2 days later than high risk participants in a control group who were not asked these questions. Hence, asking about suicidality apparently conferred short-term benefits to those at most risk. In support, Mathias and colleagues [25] in a sample of mainly 14 year olds with experience of in-patient psychiatric care reported a dose–response effect where adolescents with greater severity of suicidal ideation reported greatest reduction in ideation in repeated assessments over 6-month intervals [25]. These studies are important in establishing the impact of participation in research over time for young samples, albeit in research focused on suicide or with clinical groups. Notably, within self-harm research, the potential salutary effects of study participation over time for the most vulnerable was supported in a University-based sample over a 3 week period [24], but not in a school-based sample over a 1-year period [20]. Hasking and colleagues [20] demonstrated that a deterioration in psychological functioning over time (i.e. increased vulnerability) was associated with a change in evaluation of study participation from a positive to a negative valence at 1-year follow-up. Given that clinical decisions may often be based on short-term assessment of risk—hours, days, weeks, rather than years—short-term follow-up studies may improve the clinical relevance of study data [26, 27]. It is therefore important to test the impact of participation in a self-harm study with a school-based population using a short-term prospective design. Such prospective examination will also be important in establishing if school-based youth with and without self-harm experience differ in their response to repeated assessment. Of note, Muehlenkamp and colleagues [28] found that University participants without self-harm experience were less amenable to repeat participation.

### Current study

The current study sought further understanding of how school-based adolescents with and without experience of self-harm felt about taking part in a longitudinal study about self-harm. Specifically, the impact of study participation on early adolescents (aged 15 years and under) was sought. Other self-harm/suicide studies that have included youth of this age have predominantly targeted participants across a broader span of adolescence [19–21, 25]. Given evidence that the pattern of risk for adolescent self-harm may differ in early, mid and late adolescence it is important to distinguish between these developmental stages [14, 15]. As male and female respondents have been shown to differ in response to research participation [21], and are known to differ in prevalence of self-harm [15] a nuanced examination of responses to participation based on gender and self-harm status was also sought. Given that prospective studies with short follow-up phases are recommended for clinically relevant research [26, 27], this study seeks to evaluate the impact of asking young people to take part in a longitudinal study over a short time period (10–12 weeks) and strike a balance between being sufficiently short-term to enable clinical relevance, but also sufficiently spaced in time to be accommodated within a dense school timetable. Recent research has recommended taking steps to reduce any potential negative impact of study involvement on youth [21]. Mood elevation techniques have been employed following lab-based self-harm research [28, 29] and studies using other methods [7, 30] and are also recommended in online settings [24, 31]. An additional aim of the present study was to evaluate the use of a simple mood elevation tool that can easily be incorporated into a paper-based survey. A multi-method exploratory approach combined quantitative and qualitative analysis to augment understanding and maximise interpretation of findings [32]. Specifically the present research asked (1) Does participation in a longitudinal self-harm survey have an impact on participant mood? (2) How do young people rate and describe their experience of participation? (3) Do young people engage with a simple mood elevation device following participation in a self-harm survey? As our multi-method examination is largely exploratory no testable predictions were made. Responses across these outcomes (mood impact/survey rating/survey description/engagement with a mood elevation device) were compared for the sample overall and according to self-harm status and gender.

## Methods

### Participants

Participants were recruited from three secondary schools in the East Midlands of England to a broader study on impulsivity and self-harm. The study ran from October 2016 until February 2017. Parents of students in years 9 and 10 (aged 13–15 years) were sent an information sheet and opt-out consent form via electronic parent mail and asked to discuss the study with their child. School assemblies and tutor sessions, held before data collection, reinforced information and participant rights. Reminder messages were sent to parents 1 week before data collection.

A total of 710 students were invited to take part. Parental consent was withdrawn from n = 18 (2.5%). In addition, 46 students (6.5%) did not take part due to withdrawing assent (n = 11), other school commitments, or absence. The total number of participants completing the survey at baseline was thus 646. Recruitment was spread across schools (198:218:230). The mean age of participants was 13.5 years, (SD = .61) and 94% of the sample were aged 13–14 years. The sample was 51% male, 46% female, with 3% not stating a gender. The majority (81%) identified their ethnicity as white. Of the baseline participants, 594 completed the follow-up survey. Average follow-up time was *12.1* *weeks*, *SD* = *1.15*. The retention rate of 92% compares favourably with other school-based longitudinal studies [21]. Reasons for attrition (n = 52) at follow-up included spoiled or missing codes from completed papers n = 27 (52%); parent removed consent for follow-up n = 3 (5.7%); and unspecified absence n = 22 (42%). Distributions of gender (male 50%, female 47%, 3% unspecified) and ethnicity (white 84%) were similar at follow-up. Main analysis focuses on those who participated at both time points.

### Materials and measures

#### Questions about self-harm behaviour

Participants were provided with a definition of self-harm based on NICE (National Institute for Health and Clinical Excellence) guidelines [33]: “Self-harm is hurting yourself on purpose such as cutting, hitting, biting, burning or self-poisoning (such as swallowing too many pills or other dangerous substances), *no matter what the reason.* Self-harm is not hurting yourself by accident.” This definition reflects a lack of categorical distinction between self-harmful behaviour with or without suicidal intent [34]. Participants were asked two questions modified from the Lifestyle and Coping Questionnaire [LCQ: 2]: “Have you ever seriously *thought* about trying to harm yourself on purpose in some way but *not* actually done so?” and “Have you ever on purpose harmed yourself in some way?” A modified version of the LCQ has been used in other school-based studies [35]. Analyses for the present study are based on answers to the two self-harm questions indicated above. However, the full survey included a number of additional questions relating to self-harm which asked participants for information about how recently and frequently they self-harm; to provide a description and reason for their most recent episode; and to quantify the typical length of time between first having the urge to self-harm and completing the act. Participants were also asked two questions about help-seeking behaviour in school. All participants were asked to provide an answer to the self-harm questions, even if this was to write “not relevant”. This ensured that all participants completed each section and sought to reduce the visible distinction between those with and without experience of self-harm during testing.

#### Current mood rating scale

Participants were asked to rate current mood state on a visual analogue scale (VAS) at the start and end of the survey. This approach has been used in qualitative self-harm research with adolescents [36]. The VAS had response options ranging from 0 (illustrated by a sad face and additional text “I feel really sad and down in the dumps”) to 10 (illustrated by a happy face and “I feel really happy”). At the midpoint a neutral face and the words “I’m not feeling happy or sad” represented a score of 5. Participants were asked to mark their current mood on the scale. Comparison of pre- and post-survey VAS ratings provided an estimate of the immediate emotional impact of participation.

#### Survey rating

Participants were asked to rate their experience of taking part in the survey by selecting from provided response options, which were positively-valenced (interesting, enjoyable); negatively-valenced (upsetting, annoying); or neutral (fine), or by supplying their own term of reference in an open-response section. Multiple response choices were not prohibited.

#### Open questions about the survey

An open response question asked participants to “Describe your thoughts about taking part in the survey and any feelings the content may have raised”.

#### Doodle activity page

The final survey page contained cute animal images, cartoons, exam howlers, jokes, a space to write a joke, and doodle/colour-in spaces. New doodles and imagery were included at follow-up to maintain interest and novelty. Participants were invited to engage with this page once they had completed the survey, or wished to withdraw, with the following invitation: “The survey has now finished. Thanks for taking part! Time to chill… Check out the following page.” “Engagement” was defined as a demonstrable sign of actively engaging with the activities and spaces on the doodle page by drawing/doodling/colouring in/writing on the page etc. This page aimed to recalibrate mood, which may have been lowered through participation. Evidence suggests that looking at cute images of animals, cartoons and emotive texts are effective at eliciting positive mood [37, 38].

### Procedure

Ethical approval was obtained from the Division of Psychiatry and Applied Psychology Research Ethics sub-committee at The University of Nottingham. All survey materials were trialled, piloted and modified with a youth advisory panel with lived experience of self-harm. On the day of the baseline study consented students were provided with an information sheet, assent form and envelope. Study procedures, rights of withdrawal and limits of confidentiality and anonymity were explained by the researcher (in person or by video) or by individual tutors according to a set script. Participants generated a unique identification (ID) code and wrote this on their survey. In order that surveys could be linked to a student if responses indicated concern for safety, students were asked to include their ID code on a signed assent form and envelope, and to seal the form inside the envelope. Sealed envelopes and surveys were collected and stored separately. Procedures were repeated at follow-up. Data collection took place during designated lesson time. Students sat individually within class groups and were instructed not to discuss answers. All students received a resource sheet detailing sources of support in school and appropriate outside agencies. Survey responses were screened within 24 h of data collection for safeguarding reasons.

### Analysis approach

Data were analysed using SPSS v24 for Windows. Paired sample T tests were used to examine differences in mood scores pre- to post-survey at baseline and at follow-up for the sample overall. Between-subjects ANOVAs were used to examine effects of self-harm status (yes—a reported history of self-harm vs. no—no reported history of self-harm) and gender (Boys vs. Girls), and the gender*self-harm status interaction, for influence on mood-change scores (post VAS score–pre VAS score) at baseline and follow-up. For statistically significant interactions, simple main effects and pairwise comparisons were examined using a corrected p value to control for multiple comparisons (*p* = *.025*). For non-significant interactions, main effects analyses were performed. Chi square analysis was used to compare distributions of categorical ratings of the survey (positive/negative/neutral)—these were compared for those with and without lived experience of self-harm at baseline and follow-up. Analysis of standardised residuals identified where observed ratings in each category differed from those expected by chance (positive or negative residuals > 1.96). Qualitative responses were coded using thematic analysis [39]. Thematic analysis is a flexible form of pattern recognition which allows themes to be derived inductively (from the data) and deductively (from past literature and theory) in order to best capture and summarise a phenomenon of interest. A sample of transcribed responses were independently read and coded inductively by JL and LR. A coding frame that integrated inductively- and deductively-derived codes was then developed by JL, verified via discussion, and applied to the full data set. The coding frame contained labels, descriptions and examples of codes and themes [40]. Themes were identified and refined into main themes and sub-themes. A third researcher blind to study aims independently tested the applicability of data-to-theme allocation from randomly selected extracts with percentage consensus agreement of 83%. Consensus of 70% or above is deemed necessary for themes to be judged as coherent and valid [40].

## Results

### Initial analysis

#### Completers v non-completers

Initial analysis compared the 594 participants who completed both the baseline and follow-up surveys (completers) with the 52 who only provided baseline data (non-completers). Chi square tests revealed that groups did not differ by gender (*p* = *.287*) or ethnicity (*p* = *.497*). However, groups differed according to school (*p* < *.001*). Groups did not differ in terms of self-harm incidence (*p* = *.313*); or thoughts (*p* = *.121*). Nor were they more likely to have rated the survey at baseline as a negative rather than a positive experience *(p* = *.734*). Mann–Whitney U tests revealed no difference between groups in the distribution of mood-change scores pre- to post-survey (*p* = *.367*).

#### Incidence of self-harm thoughts and behaviour

At baseline, 30.4% of participants indicated having had thoughts of self-harm and 23.6% indicated lifetime self-harm. At follow-up, rates of self-harm thoughts were similar to baseline (30.6%), and reported incidence of lifetime self-harm was 27.6%. Of the additional 29 respondents indicating self-harm behaviour at follow-up, 25 reported first onset of behaviour between the baseline and follow-up assessment.

### Did current emotional rating scores change following completion of the survey?

A 2 × 2 between subjects ANOVA revealed a statistically significant interaction between gender and self-harm status on mood-change score from pre to post survey completion at baseline *F*(*1*,*467*) = *4.673*, *p* = *.031*, *partial η*^2^ = .*010*. Simple main effects analysis revealed there was no significant overall effect for self-harm status *(p* = *.755*); however, there was an overall statistically significant difference in mean mood change scores by gender. Specifically, mood change scores differed between boys with a self-harm history and girls with a self-harm history, *F*(*1*,*467*) = *8.189*, *p* = *.004*, *η*^2^ = *.017* (Bonferroni corrected). There was no significant difference between boys and girls who had not self-harmed (*p* = *.447*). Table 1 presents mean VAS scores at both baseline and follow-up for boys and girls with and without self-harm, and the complete sample. Findings suggest that completing the survey had a negative impact on mood for girls who had self-harmed (post-survey mood scores were lower than pre-survey scores), but conversely a positive impact on mood for boys who had self-harmed (post-survey scores were higher than pre-survey scores). A second ANOVA compared mood change scores pre-to-post survey for boys and girls across levels of self-harm status at follow-up. This time there was no statistically significant interaction between gender and self-harm status *F*(*1*,*427*) = *.379*, *p* = *.538*, *partial η*^2^ = *.001*. Main effects analysis revealed no statistically significant main effect of gender *F*(*1*,*427*) = *1.278*, *p* = *.259*, *partial η*^2^ = *.003*; or main effect of self-harm status *F*(*1*,*427*) = *.021, p* = *.884*, *partial η*^2^ = *.000*. Hence, neither gender nor self-harm status influenced mood change scores at the follow-up timepoint (See Table 1).

**Table 1:** Mean pre-survey and post-survey mood scores at baseline and follow-up

Self-harm status	Gender	Baseline			Follow-up		
		N	VAS pre-	VAS post-	N	VAS pre-	VAS post-
SH no	Boys	199	7.09 (1.82)	7.21 (1.99)	176	7.03 (1.89)	6.72 (2.24)
	Girls	164	6.72 (1.86)	6.68 (2.15)	138	6.67 (1.76)	6.67 (2.01)
SH yes	Boys	43	5.93 (2.29)	6.35 (2.28)^a^	45	6.12 (2.22)	5.48 (2.44)
	Girls	65	4.97 (1.77)	4.79 (1.85)^a^	72	5.33 (2.13)	4.58 (2.24)
Overall		491	6.60 (1.97)	6.54 (2.18)	489	6.49 (1.9)	6.22 (2.3)^b^

The table presents means for the VAS (visual analogue scale) ratings provided at the start (VAS pre-) and at the end (VAS post-) of each survey assessment for the sample overall, and by self-harm Status and Gender. Standard deviations are shown in parentheses

“SH yes” denotes lifetime incidence of self-harm. “SH no” denotes no reported history of self-harm

^a^ A significant interaction between mean mood-change score for boys and girls at the level of SH yes *F(1467)* *= 8.189*, *p = .004*, *η*^*2*^ *= .017* which survives Bonferroni correction at p = .025

^b^ A statistically significant difference between VAS pre- and VAS post-survey scores, *t* *= 3.807,* *p* *<* .*0001*

### How did participants rate the survey?

Table 2 presents proportions of participants rating each survey in positive (“interesting”, or “enjoyable”), neutral (“fine”), and negative (“annoying” or “upsetting”) terms. Most participants at baseline rated the survey in positive/neutral terms overall (79.7%) and across gender and self-harm status. However, comparing groups by self-harm status: Chi square analysis revealed that the ratings differed between those with and without self-harm *χ*^2^ *(2)* = *37.606, p* < *.001.* Inspection of standardised residuals revealed that those who did not endorse self-harm had lower levels of negative ratings than would be expected by chance; while those with self-harm experience had higher levels of negative ratings, and lower levels of positive ratings than would be expected by chance. The most common negative responses cited by those without lived experience of self-harm were “annoyance” (n = 17, 4.3%) and “boring/pointless” (n = 13, 3.3%). By contrast, the most common response for those endorsing self-harm was feeling “upset” (n = 23, 16%) with a few respondents reporting finding the survey annoying (n = 9, 6.3%) or “boring/pointless” (n = 4, 2.8%). However, it is important to note that most participants did not report negative responses. Comparing ratings by gender did not reveal a significant difference in response (*p* = *.184*).

**Table 2:** Proportions of participant ratings for positive, neutral and negative evaluation of the survey at baseline and follow-up

	Baseline					Follow-up				
	N	Positive (%)	Neutral (%)	Positive/neutral (%)	Negative (%)	N	Positive (%)	Neutral (%)	Positive/neutral (%)	Negative (%)
Overall	582	170 (28.6)	309 (52.0)	479 (79.7)	103 (17.3)	578	136 (23.5)	300 (51.9)	436 (73.5)	142 (23.9)
SH yes	119	25 (18.5) −	64 (47.4)	183 (60.6)	46 (34.8) +++	155	30 (19.4)	77 (46.5)	107 (69.0)	48 (31.0)
SH no	439	145 (32.6)	240 (55.3)	391 (86.1)	54 (12.1) −−	423	106 (25.1)	223 (51.3)	329 (77.7)	94 (22.2)
Girls	273	73 (26.7)	147 (49.0)	220 (76.2)	53 (19.4)	270	60 (22.2)	148 (54.8)	208 (77.0)	62 (23)
Boys	293	96 (32.8)	153 (52.2)	249 (84.3)	44 (15.0)	292	74 (25.3)	147 (50.3)	221 (76.0)	71 (24.3)

−/+ Standardised residual score of > 1.96; − −/++ standardised residual score of > 2.58; − − −/+++ standardised residual score of > 3.29 at p < .01 (.05/5)

“SH yes” denotes lifetime incidence of self-harm, “SH no” denotes no reported history of self-harm

At follow-up, the survey was again rated in positive/neutral terms by the majority overall (73.5%) and across self-harm status and gender. However, an increased percentage of respondents gave the survey a negative response at follow-up, compared to baseline, and this was driven in part by an increase in those finding the survey “boring” or “pointless” (8.7 v. 3.1% at baseline). Chi square analysis revealed that the distribution of positive, negative and neutral ratings did not differ according to self-harm status (*p* = *.071*). The most common negative response cited by those without self-harm was “boring” (increased to 10.4% from 3.3%) with “annoying” selected by an increased 6.9% compared to 4.3% at baseline. Similarly, the most common response for those with self-harm was now “annoying” (14.2%) with feeling “upset” reduced from 16 to 10.3%. Notably, for those endorsing self-harm the percentage of negative evaluations was lower at follow-up than at baseline while positive evaluations were proportionally higher at follow-up; the opposite pattern of response was reported in those without self-harm experience for whom positive ratings decreased and negative ratings increased in comparison to baseline. Of the 25 participants who revealed a first incidence of self-harm between assessments, most rated the survey as a positive/neutral experience at baseline (83%) and follow-up (60%), although again the response pattern reflected an increase in negative ratings by follow-up, and the highest proportion of negative response for any category of respondent. Again, when comparing ratings by gender, no significant difference in response was observed at follow-up (*p* = *.545*).

### What did participants think about taking part in the survey?

**Fig. 1:**
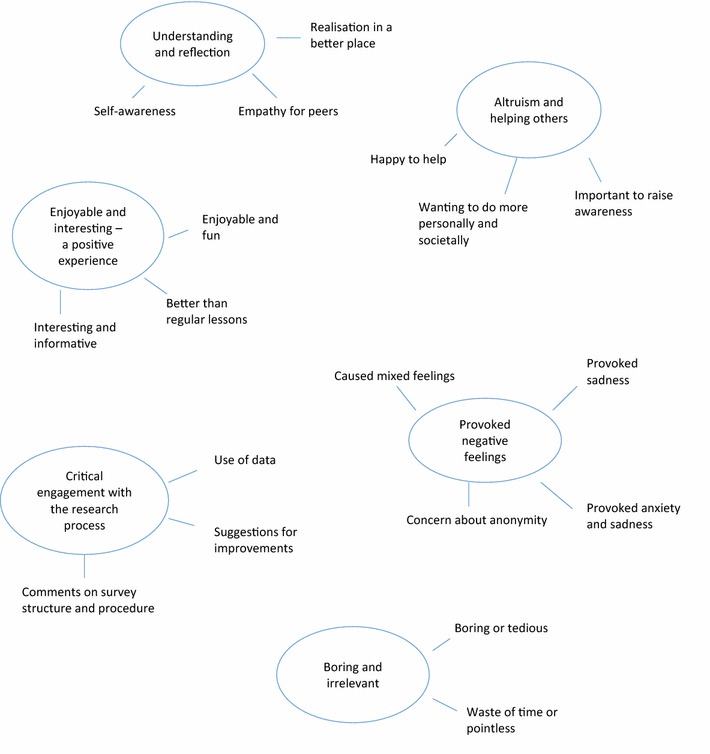
Thematic map showing six main themes (circled) and subthemes reflecting participant views on taking part in the research

Responses to the item “Please share your thoughts about taking part in the survey, and any feelings the context may have raised” were refined into six themes (three positive, two negative and one neutral) using thematic analysis [39]. No main thematic differences emerged between time-points. Main themes, subthemes, and frequencies of endorsement are shown in Fig. 1.

#### Theme: Understanding and reflection

Young people valued the greater self-awareness and understanding gained from participation: *“It’s a really good and interesting way to gain information and think about your life.”* (F, aged 14, SH). Participants felt that they “knew themselves better” from the experience and enjoyed the opportunity for self-reflection: *“I think it* [taking part] *brings you more in touch with your feelings and allows you to get presence and really think.”* (M, aged 13, no SH). For some it was greater understanding of others that was important: *“It makes me more aware of the emotional health of my peers.”* (F, aged 13, no SH.) Taking part was a chance to offload and also provided relief: *“It’s made me feel relieved that I have let out how I feel”* (F, aged 13, SH). Some found value in realising they were in a good place: *“I realise now that I enjoy lots of things and I am a better and happier person that I used to be.”* (F, aged 13, SH); *“It’s just reminded me how much happier I am now than when I was so sad, so that’s good.”* (F, aged 15, SH). This theme was the most consistently endorsed overall with endorsement from 50 participants at baseline (28% of responses) and 30 participants at follow-up (18% of responses). Overall, a slightly higher numbers of girls (n = 44) than boys (n = 36) endorsed this theme.

#### Theme: Altruism and helping others

Being able to help others was a source of value: *“I hope my input will help people for the better.”* (F, aged 13, no SH); *“It’s ok, and didn’t upset me and I’m happy to help.”* (M, aged 13, SH). The benefits were often linked to contributing to research*: “I feel happy I have taken part in some useful research.”* (F, aged 13, no SH). Students felt it was important to raise awareness of mental health: *“I think that it is good that people are recognising that mental health in young teenagers, especially students, is a big deal.”* (F, aged 14, SH). Some wanted further opportunities and support to discuss such issues: *“I think we should get lessons in PSHE* [Personal, Social and Health Education] *about self*-*harm and depression and suicide as it is a bit of a stigma topic and it shouldn’t be.”* (F, aged 14, no SH). A number of students felt that schools could do more to facilitate peer support: *“I don’t know how to help people who self*-*harm and feel that this is something that schools should teach.”* (F, aged 13, no SH). This was the second most consistently endorsed theme overall, endorsed by 33 participants at baseline (18.5% of responses) and 28 participants at follow-up (17% of responses). Endorsement was similar overall between boys (n = 31) and girls (n = 30).

#### Theme: Enjoyable and interesting—a positive experience

For some participants the process of taking part in the research was enjoyable in itself: *“I thought it was quite fun, like Christmas!”* (F, aged 13, no SH). *“It was good, I would do it anytime”* (M, aged 13, SH). For others there were additional perceived benefits, like missing class: *“Don’t mind, gets us out of lessons.”* (M, aged 13, no SH). Students felt happy to have been asked their opinions: *“I think it is good that people are researching our age group and giving us a say*.” (F, aged 14, SH). Some were pleased to be involved with a University study: *“I think it is cool that the University is asking us.”* (F, aged 13, no SH). Participants reported enjoying the survey in similar numbers at baseline (n = 26, 15%) and follow-up (n = 27, 16%). More girls than boys endorsed this theme at baseline (n = 17 vs. n = 9), a pattern reversed at follow-up (n = 12 girls vs. n = 15 boys).

#### Theme: Provoked negative emotions

Some students indicated that thinking about self-harm in others made them feel sad: “*I find it quite upsetting to know that people can feel some of the options.”* (F, aged 15, no SH). For some, the survey was a difficult reminder of past actions: “*It made me feel upset, because I remembered that time.”* (F, aged 13, SH). However, this was often a mixed emotional response: *“I felt upset because it reminded me of what I used to do, but happy because I have passed that stage in my life.”* (F, aged 13, SH). Some voiced feelings of anxiety, particularly about anonymity and confidentiality*: “I feel really anxious and in a panic because anyone could read this.”* (F, aged 13, SH). This theme was endorsed by similar numbers at baseline (n = 24, 13% of responses) and follow-up (n = 23, 14% of responses). Notably, at both time points, more girls than boys endorsed this theme—(n = 22 vs. n = 2) at baseline and (n = 17 vs. n = 6) at follow-up.

#### Theme: Boring or irrelevant

Some participants simply found the survey to be “pointless” or a “waste of their time”. Feelings that the survey was “boring”, or “repetitive” were increasingly cited at the follow-up assessment: *“Boring because we have already done it.”* (M, aged 13, no SH). For some, the lack of personal relevance was a source of annoyance: *“It’s annoying as it is not relevant and depressing.”* (F, aged 14, no SH). A small number of participants endorsed this theme, with 6 participants at baseline (3% of responses) and 12 participants at follow-up (7% of responses). This response was predominantly a male phenomenon with all but two references to boredom or irrelevance coming from boys.

#### Theme: Critical engagement with the research process

Participants offered thoughts on how the research could be improved. Some suggested that the survey did not go far enough: “*The questions were very clear, but needed more depth*.” (M, aged 14, no SH), or had, “*surprisingly little content about self*-*harm*” (M, aged 13, no SH). Others felt the survey should have included broader questions on “drugs and alcohol” or “sexuality”. Some queried what would happen with their data: “*It would be interesting to see what research you would do with the results, or what solutions you would have to problems.”* (M, aged 13, no SH). Some questioned the validity of a survey: “*I think that people who have self*-*harmed wouldn’t say it on a survey because if you self*-*harm you don’t tell anyone.”* (F, aged 13, no SH). Others wondered whether participants would be able to adequately assess their responses: *“People may not be able to evaluate what they think.”* (F, aged 13, SH). This final theme was the most commonly identified response at follow-up, with endorsement rising from 17 participants (10% of responses) at baseline to 34 participants (21% of responses) at follow-up. More boys endorsed this theme than girls overall, although proportions were similar at each time point (n = 10 boys and n = 7 girls at baseline; n = 19 boys and n = 15 girls at follow-up).

### Did participants engage with the final doodle page?

Just over half of the participants (55% baseline and 60% follow-up) chose to tangibly engage with the doodle page (e.g. doodled, filled in speech bubbles, offered a joke). At baseline a higher proportion of participants with self-harm engaged (76%) than those without (55%), but this was not a significant difference *χ*^2^ *(2)* = *2.303*, *p* = .*129*. At follow-up by contrast, a significantly higher proportion of those without self-harm (63 v 50%) tangibly engaged with this page, *χ*^2^ *(1)* = *8.045*, *p* = *.005*. There were no differences in proportions of interactions with the doodle page between boys and girls. The distribution of mood-change scores (pre- to post-survey) differed between those who did and did not complete the final activity page at baseline (Mann–Whitney *U* = *26*,*139*.*5*, *z*-*2*.*570 p* = *.010*). Those engaging with the page reported a small decrease in emotional rating (mean change in score − .19), while those not engaging reported a small increase in emotional rating (mean change in score + .05). However, distributions did not differ at follow-up (*p* = *.294*). Students commented on the final doodle page in the open response section: *“I’m rating the survey a 10 because of the cats”* (Did not say, aged 13, no SH). “*I love doing these surveys. I feel relieved to write down how I feel and I love the doodle page at the end!”* (F, aged 13, SH thoughts). A number of young people suggested that the final page had made them feel better: *“I feel strange, nervous, also confused and hurt, but relieved. Thanks for the doodles* – *it helped calm me down”* (F, aged 13, SH).

## Discussion

Overall, the present findings suggest, that for the majority, participation in research on self-harm was not perceived as a negative experience by young adolescents and did not impact negatively on mood. Participants described important benefits such as increased self-awareness, a chance to off-load, and helping others. However, subtle differences were observed according to gender, self-harm status and across time-points. Firstly, emotional rating (VAS) scores indicated that, following participation, respondents largely rated their mood at the positive (happy) end of the scale. But there were notable differences between the most vulnerable boys and the most vulnerable girls in their immediate emotional reaction to participation, as indicated by the VAS. For boys with self-harm, participation led to an improvement in mood; whereas for girls with self-harm, participation led to a deterioration in mood. The finding that high-risk boys found a mood-based benefit from involvement resonates with some previous studies [19, 24, 25] which indicate that participation can confer benefit for those at greatest risk. Although notably, this pattern of findings was not supported at follow-up. These findings suggest however, that in terms of immediate emotional reaction, conferred benefits are less likely to be found for girls who self-harm. As such, studies may need to be particularly alert to the immediate emotional impact of research participation on vulnerable girls.

The survey rating data revealed that the majority of participants judged taking part as a positive/neutral experience at both baseline and follow-up. Positive/neutral evaluations far outweighed negative evaluations for boys and girls and those with and without self-harm at both time points. Closer analysis at baseline revealed significant differences in the pattern of emotional responses felt between those with and without self-harm experience: a higher proportion of those endorsing self-harm found participation to be a negative experience and a smaller proportion rated the survey positively compared with those who did not self-harm. This suggests an increased vulnerability in response for those with lived experience of self-harm. However, differences in response distributions between these groups were not observed at follow-up. In most cases, at the second assessment, participants reported fewer positive/neutral evaluations and more negative reactions to the survey (which may be in line with the overall VAS follow-up findings) but there was one notable exception. For those endorsing self-harm, a larger proportion found the survey to be a positive or neutral experience at the second compared to first time of assessment, and negative reactions to the survey for this subset actually decreased over time. This resulted in a smaller percentage point difference in positive/neutral ratings and negative ratings between those who had and had not self-harmed. The finding of an increased positive outcome over time for those at higher risk of self-harm again chimes with previous research [25, 28] suggesting that those at greatest vulnerability may gain greatest long-term benefit from on-going participation.

The contrasting responses found from those with and without self-harm experience across VAS and survey ratings may relate to the perceived relevance of the survey for individual respondents. At follow-up, an increased number of negative reactions to participation for those not endorsing self-harm related to boredom, a lack of personal bearing and annoyance at being asked to complete a survey twice—findings which were supported in the qualitative analysis. These reactions featured far less for those with lived experience of self-harm. Relevance may drive the benefit gained from longitudinal engagement with this topic, although this does not rule out finding the survey emotionally impactful (as demonstrated by lower VAS scores). Qualitative findings suggest the increase in positive ratings at follow-up in part may relate to a possible therapeutic benefit derived from an on-going opportunity to “offload” and self-reflect. This may be particularly important for groups typically unlikely to have disclosed their behaviour [2] or lacking opportunity to discuss and describe it. It could also be argued that exposure to the topic at baseline may have desensitised participants for the follow-up assessment. The effects of this could be greatest for those with lived experience who may have felt a greater emotional response to the topic at the outset. The sharp increase in negative evaluations of the survey for those without lived experience at follow-up suggests it will be important for future research to explore the impact of research participation for those who are psychologically healthy, as well as those at greater risk, over repeated assessment, particularly where follow-up is relatively short. In particular, increased rates of annoyance mainly for those not endorsing self-harm behaviour (see also [28], but also across the sample overall, should be recognised and mitigated where possible.

The findings also highlight the varied nature of individual response to participation. Engaging with a sensitive topic may cause understandable distress for some (such as the lowering of mood found for girls with self-harm), but it does not necessarily follow that this is evaluated as a “negative” outcome. Markedly, many participants coupled positive and negative ratings, separating emotional responses from a cognitive evaluation (e.g. *nervous* yet *interesting; uncomfortable,* but *fine; difficult* yet *worthwhile).* Given the complexity of the behaviour, it is not surprising that respondents selected multiple categories to describe their response. This suggests that it is important for ethical guidelines around self-harm research to recognise that potential benefits and potential risks from involvement are not necessarily mutually exclusive.

Although there was no statistical distinction between boys and girls when comparing survey ratings, analyses indicated differences in emotional response to survey participation according to both VAS scores and thematic analysis, where a qualitatively different reaction to survey participation from girls, who did describe feeling upset, was found to boys, who broadly did not. Further qualitative research may help to clarify these gender differences in response to participation. The qualitative findings largely support those found by Hasking and colleagues [20] in their school-based sample. A novel thematic finding in this study was the large endorsement for a critical engagement in the research process indicating that many young people are not only supportive of research endeavour but are keen to reflect on, question and challenge the process.

This study also provides insight into the use of a simple mood recalibration doodle page. A small majority of participants chose to engage with this page, though rates of engagement varied across groups. At baseline, those whose mood decreased the most (participants endorsing self-harm) had a higher rate of engagement with the page. At follow-up, those who reported an increase in negative survey ratings (participants not endorsing self-harm) were more likely to demonstrably engage. It could be argued that those feeling the greatest negative impact from participation may more readily seek out recalibration, but more work should seek to evaluate the impact of such mitigation tools in community samples using longitudinal designs. The present study did not provide an experimental test of mitigation or specifically elicit participants’ reactions to the doodle page. We can not know to what extent the page was helpful for those who nonetheless left no physical indication of engagement. However, large numbers of participants did demonstrably engage and many chose to reference this in open responses. Undoubtedly for some, the page helped to calm emotions. Moreover, the study’s advisory youth panel strongly endorsed the doodle page. Importantly, the page brought an additional and unexpected ethical advantage. The self-penned jokes, doodles, or direct comments written directly on the survey script by participants who also used the page to offer reassurance to the research team that they were feeling all right, had a positive impact on researcher wellbeing. Collecting data on self-harm has an inevitable impact on researchers but the evaluation of this impact is under-researched. The need to better document and discuss harm minimisation for researchers has been discussed elsewhere [31, 41] and sharing potential practical solutions is advocated.

Key strengths of this study include the focus on a community-based sample of early adolescents (aged 13–14) for whom self-harm risk is heightened [15] and the additional insight offered on how both male and female participants, with and without self-harm experience, respond differentially to study involvement. Given recommendations for short-term prospective examinations of self-harm risk in youth [26, 27] this study provides important ethical encouragement, via multiple and converging methods, that short-term assessment (at least in terms of weeks) does not confer added risk to the majority of participants. In addition, novel insight is provided into the role of a simple mood enhancement tool. The low attrition (8%) compares favourably with previous school-based research [21]. High willingness to complete a follow-up survey may be seen as an additional marker of a study’s acceptability. Nonetheless, the influence of the school-based setting must be recognised. Schools, as an “adult-owned territory” [42] hold an inherent power asymmetry within which children generally participate in compulsory activities [43]. Thus, despite clear efforts to emphasise participant rights to withdraw, a learned compliance can compromise the voluntary principles of participation [44]. There are limitations to the conclusions that can be reached from this study. We did not explicitly ask participants at follow-up how they felt after completing the baseline assessment and we can not examine if reported reactions were transitory. Neither did we explicitly ask participants if they found the research to be worthwhile. A small number of students (4%) indicated initiating self-harm behaviour between assessment points. This compares with rates reported in other prospective school-based studies of 2.6 and 6.0% [13, 45]. While the development of self-harm observed here may follow the natural trajectory of self-harm, the design of the study does not allow us to rule out any causal iatrogenic link. These questions would be usefully addressed in future studies. The present study largely assesses self-harm in terms of a lifetime presence of behaviour. While this broad indicator of self-harm status was adequate in distinguishing differences in response, meaningful information about the impact of study involvement is likely to be gained from a finer grained analysis of self-harm status in which the recency or frequency of behaviour is accounted for. Notably, those indicating the most recent onset of self-harm (i.e. first time behaviour occurring between assessment points) recorded a high proportion of negative responses at the follow-up assessment (40%). Those with current versus historical self-harm may differ in both emotional response and cognitive appraisal of that response. Further research should explore these ideas.

## Conclusions

This study contributes important information on the impact of research participation on young adolescents using quantitative and qualitative data to augment understanding. Participation was, for the most part, reported to have been a positive and beneficial experience, and many valued the chance to critically engage with the research process. Those with self-harm experience, and in particular girls who self-harm, displayed an increased vulnerability compared to those who did not self-harm (lower mood ratings following participation, a larger proportion of negative ratings) but, nonetheless, most evaluated their participation in positive or at least neutral terms. However, further work is needed to understand the impact of repeated assessment on those with and without lived experience for whom research reactions qualitatively differ. Many young people felt that having an opportunity to discuss mental health in school was important and may confer unique benefits for those who self-harm. School settings are potentially well placed to accommodate appropriate response to risk and provide support. Ensuring that any school-based support is appropriate and effective is critical however. Evidence-based school programmes such as the Signs of Self-Injury Programme [46], for example, which are designed to educate about self-harm and offer skills to staff and students to respond to self-harm may offer a promising and systematic way forward [47]. Prospective research on adolescent self-harm is ethically viable in schools, but the inclusion of a simple mood-elevating tool may be an additional and easily incorporated means of mood elevation, and beneficial to participants and researchers.

## Abbreviations

NICE: National Institute for Health and Clinical Excellence
LCQ: Lifestyle and Coping Questionnaire
VAS: visual analogue scale
F: female
M: male
SH: self-harm
PSHE: Personal, Social and Health Education
IRB: Institutional Review Board

## References

[1] Kapur N (2013). Non-suicidal self-injury v. attempted suicide: new diagnosis or false dichotomy?. Br J Psychiatry.

[2] Madge N (2008). Deliberate self-harm within an international community sample of young people: comparative findings from the Child & Adolescent Self-harm in Europe (CASE) Study. J Child Psychol Psychiatry.

[3] Muehlenkamp J (2012). International prevalence of adolescent non-suicidal self-injury and deliberate self-harm. Child Adolesc Psychiatry Ment Health.

[4] NICE, N.I.f.H.a.C.E. (2004). Self-harm: the short-term physical and psychological management and secondary prevention of self-harm in primary and secondary care. Clinical guidelines, No. 16.

[5] Owens D, Horrocks J, House A (2002). Fatal and non-fatal repetition of self-harm: systematic review. Br J Psychiatry.

[6] Mars B (2014). Clinical and social outcomes of adolescent self harm: population based birth cohort study. BMJ.

[7] Townsend E (2016). Self-harm and life problems: findings from the Multicentre Study of Self-harm in England. Soc Psychiatry Psychiatr Epidemiol.

[8] Whitlock J (2010). Self-injurious behavior in adolescents. PLoS Med.

[9] Morey Y, Mellon D, Dailami N, Verne J, Tapp A (2016). Adolescent self-harm in the community: an update on prevalence using a self-report survey of adolescents aged 13–18 in England. J Public Health.

[10] Moran P (2012). The natural history of self-harm from adolescence to young adulthood: a population-based cohort study. Lancet.

[11] Ogle RL, Clements CM (2008). Deliberate self-harm and alcohol involvement in college-aged females: a controlled comparison in a nonclinical sample. Am J Orthopsychiatry.

[12] Whitlock J, Eckenrode J, Silverman D (2006). Self-injurious behaviors in a college population. Pediatrics.

[13] Stallard P (2013). Self-harm in young adolescents (12–16 years): onset and short-term continuation in a community sample. BMC Psychiatry.

[14] Morgan C (2017). Incidence, clinical management, and mortality risk following self harm among children and adolescents: cohort study in primary care. BMJ.

[15] Geulayov G (2017). Incidence of suicide, hospital-presenting non-fatal self-harm, and community-occurring non-fatal self-harm in adolescents in England (the iceberg model of self-harm): a retrospective study. Lancet Psychiatry.

[16] Lakeman R, Fitzgerald M (2009). The ethics of suicide research. Crisis.

[17] Lakeman R, Fitzgerald M (2009). Ethical suicide research: a survey of researchers. Int J Ment Health Nurs.

[18] Dazzi T (2014). Does asking about suicide and related behaviours induce suicidal ideation? What is the evidence?. Psychol Med.

[19] DeCou CR, Schumann ME (2017). On the iatrogenic risk of assessing suicidality: a meta-analysis. Suicide Life Threat Behav.

[20] Gould MS (2005). Evaluating iatrogenic risk of youth suicide screening programs: a randomized controlled trial. JAMA.

[21] Hasking P, Tatnell RC, Martin G (2015). Adolescents’ reactions to participating in ethically sensitive research: a prospective self-report study. Child Adolesc Psychiatry Ment Health.

[22] Langhinrichsen-Rohling J (2006). Sensitive research with adolescents: just how upsetting are self-report surveys anyway?. Violence Vict.

[23] Robinson J (2011). Does screening high school students for psychological distress, deliberate self-harm, or suicidal ideation cause distress—and is it acceptable?. Crisis.

[24] Whitlock J, Pietrusza C, Purington A (2013). Young adult respondent experiences of disclosing self-injury, suicide-related behavior, and psychological distress in a web-based survey. Arch Suicide Res.

[25] Mathias CW (2012). What’s the harm in asking about suicidal ideation?. Suicide Life Threat Behav.

[26] Glenn CR, Nock MK (2014). Improving the short-term prediction of suicidal behavior. Am J Prev Med.

[27] Franklin JC (2017). Risk factors for suicidal thoughts and behaviors: a meta-analysis of 50 years of research. Psychol Bull.

[28] Muehlenkamp JJ (2014). Emotional and behavioral effects of participating in an online study of nonsuicidal self-injury. Clin Psychol Sci.

[29] Arbuthnott AE, Lewis SP, Bailey HN (2015). Rumination and emotions in nonsuicidal self-injury and eating disorder behaviors: a preliminary test of the emotional cascade model. J Clin Psychol.

[30] Wadman R (2017). A sequence analysis of patterns in self-harm in young people with and without experience of being looked after in care. Br J Clin Psychol.

[31] Lloyd-Richardson EE (2015). Research with adolescents who engage in non-suicidal self-injury: ethical considerations and challenges. Child Adolesc Psychiatry Ment Health.

[32] Leech NL, Onwuegbuzie AI (2010). Guidelines for conducting and reporting mixed research in the field of counseling and beyond. J Couns Dev.

[33] NICE, N.i.f.H.a.C.E. (2004). Self-harm in over 8 s: short-term management and prevention of recurrence.

[34] Orlando CM (2015). Nonsuicidal self-injury and suicidal self-injury: a taxometric investigation. Behav Ther.

[35] O’Connor RC (2009). Self-harm in adolescents: self-report survey in schools in Scotland. Br J Psychiatry.

[36] Wadman R (2016). An interpretative phenomenological analysis of the experience of self-harm repetition and recovery in young adults. J Health Psychol.

[37] Nittono H (2012). The power of kawaii: viewing cute images promotes a careful behavior and narrows attentional focus. PLoS ONE.

[38] Goritz AS (2007). The induction of mood via the WWW. Motiv Emot.

[39] Braun V, Clarke V (2006). Using thematic analysis in psychology. Qual Res Psychol.

[40] Boyatzis RE (1998). Transforming qualitative information.

[41] Mckenzie SK, Li C, Jenkin G, Collings S (2016). Ethical considerations in sensitive sucide research reliant on non-clinical researchers. Res Ethics.

[42] Morrison K (2013). Interviewing children in uncomfortable settings: 10 lessons for effective practice. Educ Stud.

[43] Morrow V, Richards M (1996). The ethics of social research with children: an overview. Child Soc.

[44] Gallacher L, Gallager M (2008). methodological immaturity in childhood research? Thinking through ‘participatory methods’. Childhood.

[45] O’Connor RC, Rasmussen S, Hawton K (2009). Predicting deliberate self-harm in adolescents: a six month prospective study. Suicide Life Threat Behav.

[46] Jacobs D (2009). Signs of self-injury prevention manual.

[47] Muehlenkamp JJ, Walsh BW, McDade M (2010). Preventing non-suicidal self-injury in adolescents: the signs of self-injury program. J Youth Adolesc.

